# Case Report: Frontoparietal Metastasis From a Primary Fallopian Tube Carcinoma

**DOI:** 10.3389/fsurg.2021.594570

**Published:** 2021-02-17

**Authors:** Anthony I. Jang, Joshua D. Bernstock, David J. Segar, Marcello Distasio, Ursula Matulonis, Wenya Linda Bi

**Affiliations:** ^1^Harvard Medical School, Boston, MA, United States; ^2^Department of Neurosurgery, Brigham and Women's Hospital and Harvard Medical School, Boston, MA, United States; ^3^Department of Pathology, Brigham and Women's Hospital and Harvard Medical School, Boston, MA, United States

**Keywords:** fallopian tube carcinoma, BRCA1, metastasis, neuro-oncology, neurosurgery

## Abstract

**Background:** Metastatic brain tumors typically arise from primary malignancies of the lung, kidney, breast, skin, and colorectum. Brain metastases originating from malignancies of the female genital tract are extremely rare. We present a case of fallopian tube brain metastasis and in so doing review the pertinent literature.

**Case Description:** We describe a 59-year-old patient with a history of fallopian tube carcinoma who presented with an incidentally identified left frontal brain mass. MRI demonstrated an enhancing lesion in the left centrum semiovale with a second enhancing lesion noted in the cerebellar vermis. She underwent a left parietal craniotomy for resection of the dominant and clinically symptomatic lesion. Immunohistochemical stains were positive for PAX8 and p53, confirming fallopian tube origin.

**Conclusions:** Fallopian tube cancer brain metastasis is extremely uncommon. We highlight the treatment and surgical resection of this patient's *BRCA1* metastatic fallopian lesion and systematically review the literature regarding the pathogenesis, diagnosis, treatment, and histologic characteristics of the previously identified fallopian tube metastases to the central nervous system. The optimal course of treatment for brain metastasis of fallopian tube carcinoma has not been clearly defined due in part to the rarity of this condition. Consistent with *BRCA1* neoplasms involving the breast and ovaries, the *BRCA1* status of the patient's primary tumor likely increased the risk of central nervous system dissemination. This highlights a potential benefit of early screening of individuals with metastatic gynecologic malignancies associated with *BRCA1* in the absence of any neurological symptoms.

## Introduction

Metastatic brain tumors remain a major neurological complication of systemic cancer. They most frequently arise from primary malignancies of the lung, kidney, breast, skin, and colorectal tissue (1). Brain metastases arising from malignancies in female genital tract are rare. Fallopian tube cancer is the least-common malignant neoplasm of the female reproductive tract, comprising 0.3–1% of all gynecological malignancies (2). There are only a handful of reports of brain metastases arising from a primary fallopian tube cancer, underscoring the rarity of tubal cancers manifesting as central nervous system (CNS) metastases. This leads to difficulty in establishing standardized screening and treatment guidelines for CNS metastasis of tubal carcinoma, especially in individuals with genetic susceptibilities such as *BRCA1* that may further benefit from early screening for metastatic brain tumors. In this report, we describe a patient with a history of *BRCA1*-associated fallopian tube and breast cancer who presented with an incidentally identified left frontal brain mass.

## Case Description

**Figure 1:**
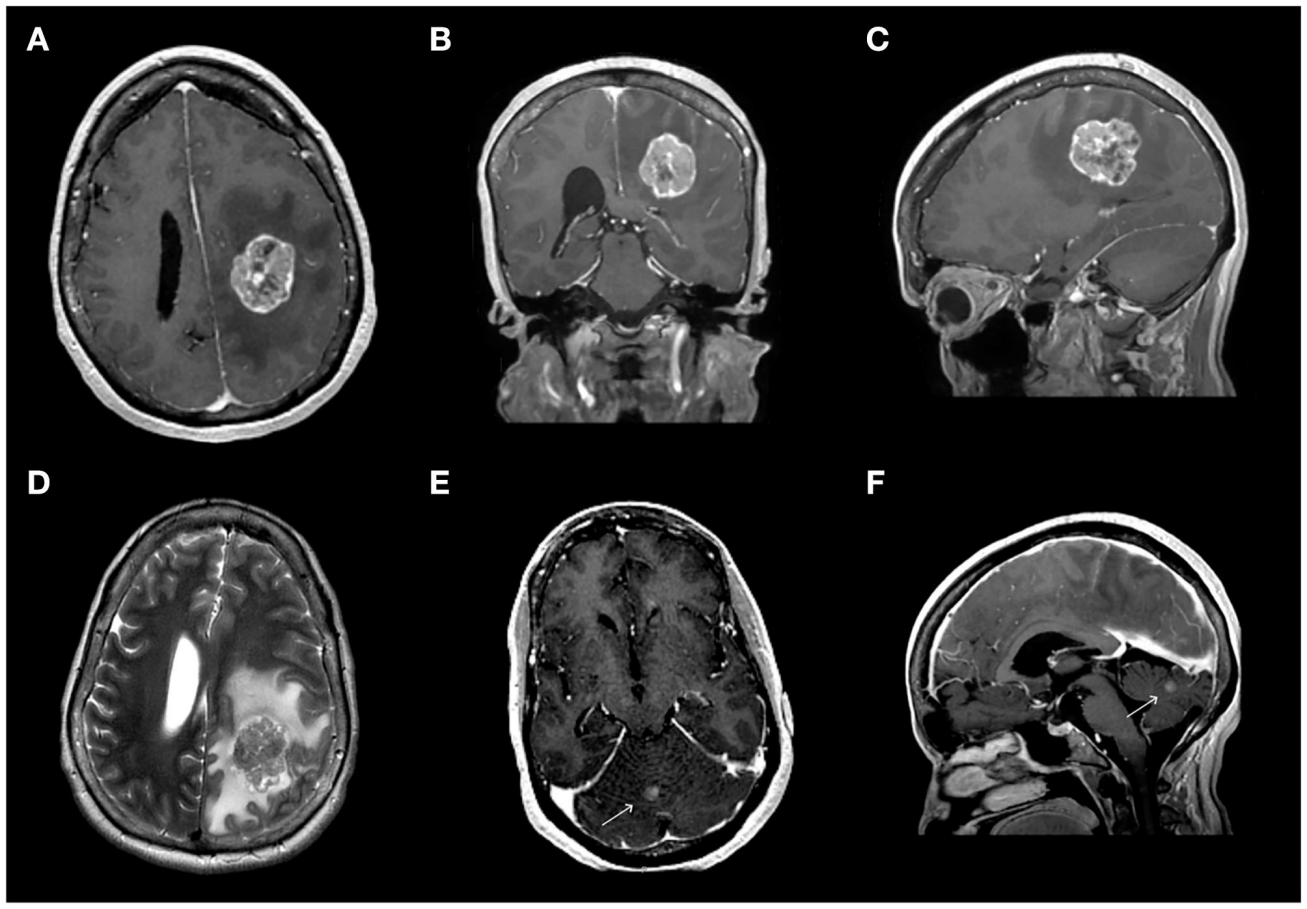
Pre-operative magnetic resonance imaging (MRI) revealed a dominant 3.7 × 2.7 × 3.7 cm left frontoparietal mass associated with diffuse edema **(A–D)** and a 6 mm lesion in the cerebellar vermis **(E,F)**. **(A–C)** Contrast-enhanced T1-weighted MR sequence showing the dominant frontoparietal lesion in the axial **(A)**, coronal **(B)**, and sagittal **(C)** views. **(D)** T2-weighted image of the dominant lesion. **(E,F)** Contrast-enhanced T1-weighted MR sequence showing the lesion in the cerebellar vermis in axial **(E)** and sagittal **(F)** views.

A 59-year-old right-handed woman with a history of metastatic breast cancer and fallopian tube cancer associated with *BRCA1* germline mutation presented after a screening head CT for a clinical trial revealed a 4 × 3.8 × 3.1 cm deep left parietal enhancing mass and a 7 × 7 × 6 mm vermian lesion. Follow-up MRI confirmed these findings and revealed diffuse vasogenic edema surrounding the frontoparietal lesion with associated mass effect on the left lateral ventricle (Figure 1). In retrospect, she had experienced several weeks of right-hand clumsiness, inability to write or type, and left-right disorientation. Neurological examination revealed mild dysarthria, right-sided dysmetria on finger-to-nose testing, mild ataxia with ambulation, and difficulty with two-part commands, with no focal weakness or numbness. Agraphia, acalculia, and left-right confusion were also noted, consistent with Gerstmann's syndrome.

The patient's past medical history is notable for ER^−^/PR^−^/HER2^+^ infiltrating ductal carcinoma diagnosed 9 years prior to presentation, for which she received 6 weeks of neoadjuvant chemotherapy with paclitaxel, carboplatin, and trastuzumab. She underwent bilateral mastectomies followed by adjuvant radiation and trastuzumab treatment. Genetic testing confirmed *BRCA1* mutant status, prompting prophylactic hysterectomy and oophorectomy, which revealed a 0.5 cm stage 1A serous carcinoma of the fallopian tube. She completed six cycles of adjuvant carboplatin and topotecan and remained disease-free for 5 years.

On annual screening of her chest and abdomen 5 years after initial diagnosis, a new right lung mass, thoracic lymphadenopathy, and liver nodules were noted on MRI abdomen. Follow-up CT chest, abdomen and pelvis confirmed the presence of subcarinal lymphadenopathy and scattered pulmonary masses. Clinically, the patient developed diffuse abdominal pain and anorexia. Lung mass biopsy confirmed metastatic serous carcinoma of fallopian origin, with immunophenotyping diffusely positive for PAX8 and p53 and negative for TTF1 and GATA3. A screening brain MRI at this time showed no evidence of any intracranial lesions. The patient initiated cediranib and olaparib on clinical trial for recurrent cancer, with disease control for 21 months. However, a subsequent screening CT scan demonstrated progressive lung disease by Response Evaluation Criteria in Solid Tumors (RECIST) criteria (3). Despite five additional months of carboplatin and doxorubicin, follow-up imaging revealed further progression of the right lung mass. Given failed salvage therapies, the patient elected to enroll in another clinical trial whose screening criteria included a head CT, prompting diagnosis of brain metastasis.

**Figure 2:**
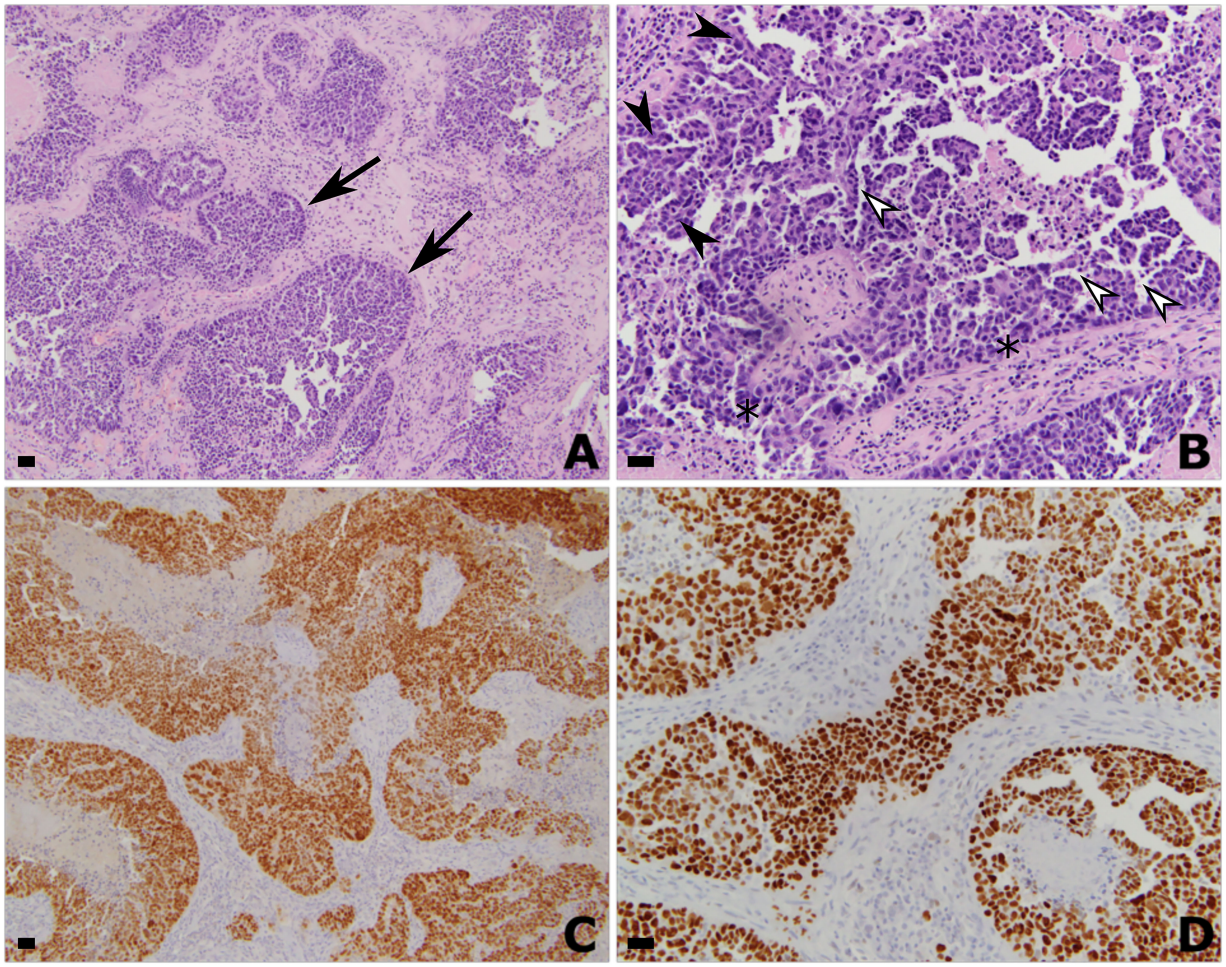
Histologic and immunohistochemistry findings of the left parietal resection specimen consistent with metastatic high-grade serous carcinoma of Mullerian origin. Hematoxylin and eosin stained histologic sections showing invasive metastatic carcinoma at **(A)** 50x and **(B)** 200x magnification. All scale bars are 50 μm. Pathology is characterized by nests of epithelioid tumor cells (arrows) with a complex glandular architecture, branching papillary fronds (filled arrowheads), slit-like fenestrations (open arrowheads), moderate to marked nuclear atypia and hyperchromasia, and frequent mitoses (asterisk). Immunohistochemistry showing strong diffuse nuclear positivity with **(C)** PAX-8 and **(D)** P53. Taken together, these findings support the diagnosis of metastatic high-grade serous carcinoma of Müllerian origin.

**Figure 3:**
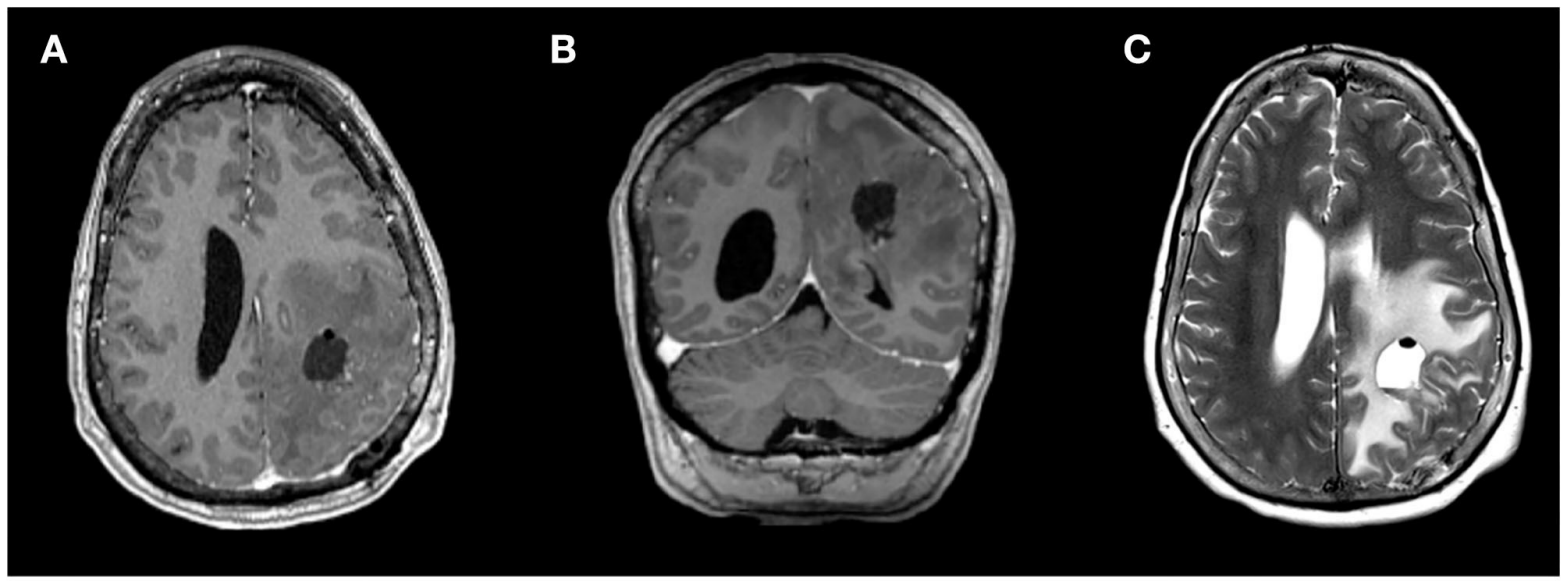
Magnetic resonance imaging (MRI) from post-operative day 1 showing gross total resection of the dominant left frontoparietal mass, on **(A)** axial and **(B)** coronal contrast-enhanced T1-weighted sequences and **(C)** T2-weighted sequence.

**Figure 4:**
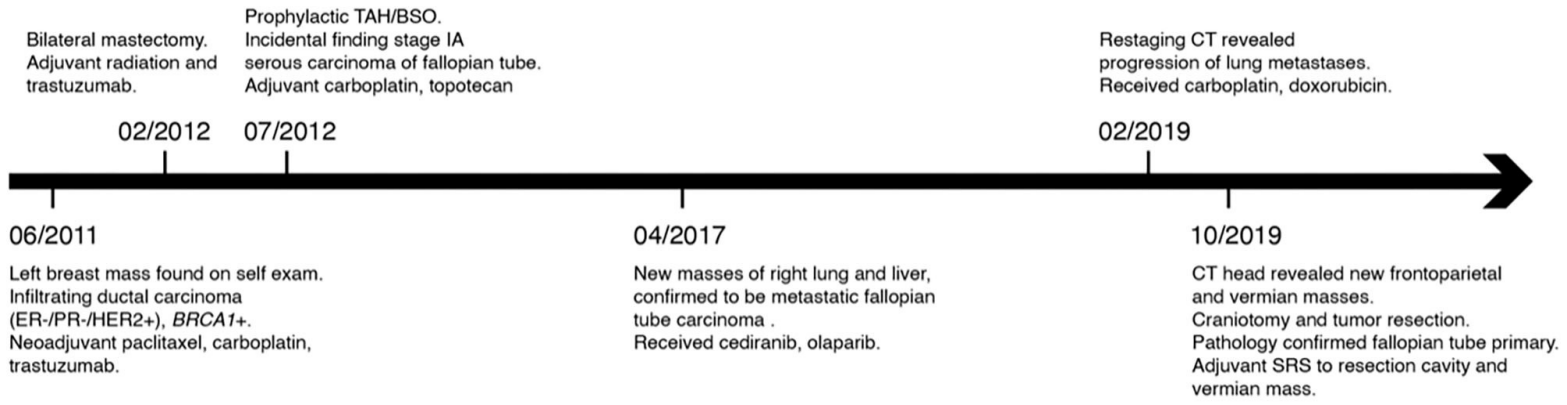
Timeline of major clinical events from initial discovery of breast cancer to craniotomy for frontoparietal tumor resection. TAH/BSO, total abdominal hysterectomy and bilateral salpingo-oophorectomy; SRS, stereotactic radiosurgery.

The patient underwent a left parietal craniotomy for resection of the dominant and symptomatic lesion. A firm, fibrous tumor was extirpated, with histopathology confirming metastatic carcinoma of fallopian tube origin (Figure 2), and gross total resection confirmed by intraoperative ultrasound and post-operative MRI (Figure 3). Post-operatively, patient remained neurologically at baseline with no notable complications. At 2 weeks after surgery, she reported improved right hand dexterity, left-right orientation, comprehension, and had regained the ability to write and type. For adjuvant brain radiotherapy, decision was made for focal stereotactic radiosurgery (SRS), reserving whole brain radiotherapy (WBRT) for salvage given her relatively young age and limited tumor volume. The patient received 16 Gy of gamma-knife stereotactic radiosurgery (SRS) to the post-operative resection cavity and 20 Gy of SRS to the second cerebellar lesion (see Figure 4 for timeline of major clinical events).

## Discussion

Fallopian tube carcinomas are rare, with an age-adjusted incidence of 0.39 per 100,000 women in the United States, and comprising <1% of all gynecological malignancies (2, 4). Furthermore, there is a low incidence of spread of a primary fallopian tube carcinoma to the CNS. Analysis of the Surveillance, Epidemiology, and End Results (SEER) database showed that only 0.21% of patients (*N* = 7,538) with primary gynecologic malignancies were found to have brain metastasis, after excluding cancers of the ovary, endometrium, and cervix. Even among the subset of individuals with metastatic disease in this cohort, 2.19% harbored brain metastases (5). The scant data available for cases such as this one highlight the challenge of determining an optimal treatment regimen for rare diseases. We identified 21 clinical trials that investigate novel therapeutic regimens for fallopian tube cancer that are currently recruiting patients and do not exclude patients with metastatic disease in the CNS (Supplementary Table 1).

Tubal cancers generally spread through the peritoneal cavity and its viscera *via* the tubal fimbria or through transmural invasion of the tubal wall. As such, the most frequent site of metastasis include the intraperitoneal surface and any neighboring organs, such as the ovaries and uterus (6). Other common sites of recurrence include the upper abdomen, retroperitoneal lymph nodes, liver, and lungs (7). Both hematogenous and lymphatic spread of fallopian tube cancers have been reported, accounting for routes to more distant metastases (8, 9). The brain as the first site of recurrent disease years after diagnosis of tubal carcinoma has been reported (10). In ovarian cancers, it has also been suggested that retrograde flow *via* the vertebral venous system may provide a potential route for metastasis to the CNS (11).

Ten patients have been reported in the literature to date with brain metastases from a fallopian tube carcinoma primary (Supplementary Table 2) (10, 12–18). The median interval between initial diagnosis of fallopian tube carcinoma and the discovery of a brain metastasis was 3 years (range 3–52 months). Sites of metastasis within the CNS varied widely and spanned supratentorial and infratentorial compartments as well as the skull base.

Surgery followed by adjuvant radiation offers improved local disease and survival advantage for oligometastases to the brain. The optimal course of treatment for CNS metastasis of tubal carcinoma has not been clearly defined due to the low incidence of this disease. Choice of treatment in prior case reports include surgical resection, radiotherapy, and combination chemotherapy mostly involving cyclophosphamide, doxorubicin, paclitaxel, and platinum-based agents including cisplatin and carboplatin. Resection was preferred for patients with a singular mass, although this was contingent on other factors including overall disease progression and baseline health (10, 17). In fact, out of the four patients with a singular brain mass, two patients did not undergo resection due to poor prognosis associated with disseminated disease. In our case, given the patient's relatively young age and good functional status, we opted for resection of her main, symptomatic frontoparietal tumor.

In the majority of reported cases, whole brain radiotherapy (WBRT) was traditionally used to augment CNS tumor control, likely reflecting the standard of practice prior to 2010 (19). In our case, the patient was treated with post-operative stereotactic radiosurgery (SRS) to reserve WBRT as an option for salvage therapy and to optimize cognitive outcomes (20, 21).

Of note, successful tumor response has been reported in a case following intra-arterial administration of chemotherapy (intra-arterial carboplatin and intravenous etoposide), suggesting that intra-arterial therapy may be an effective option for radiation-resistant CNS metastases from tubal carcinomas, and provide a potential route of treatment after tumor resection (15).

The patient in our case benefitted from an incidental finding on brain CT, highlighting the importance of screening for CNS disease in metastatic gynecologic malignancies, especially in patients with known *BRCA* mutations and disseminated disease. *BRCA1/2* carriers are recommended annual breast MRI and mammography at age 25, compared to the annual or bi-annual mammography starting age 40–50 in women at average-risk for breast cancer (22–25). *BRCA1* mutation confers an increased risk for a variety of malignancies outside of breast and ovarian cancer, including cancers of the fallopian tube, cervix, and pancreas (26, 27). Hence, bilateral salpingo-oopherectomy is generally recommended to reduce risk of gynecologic malignancies for *BRCA1* positive women who have completed childbearing (28).

Importantly, in patients with breast and gynecologic malignancies, *BRCA1* mutation also confers an increased risk of progression to brain metastasis (29–31). The incidence of CNS metastasis is significantly higher for *BRCA* mutation carriers compared to non-carriers both in overall rate (53% *BRCA1*, 50% *BRCA2*, 25% non-carriers), and rate of brain metastasis as the first event. This risk is further increased for specific tumor subtypes, such as the HER2-enriched or triple-negative breast cancer subtypes (32, 33). Consistent with this, our patient's mass was HER2-enriched and ER/PR negative. However, no generalized screening guidelines exist for malignancies of other organs such as the pancreas and brain, and therefore screening is variable based on individual risk factors and clinician preference.

This case highlights the potential importance of genetic markers such as *BRCA1* in predicting patterns of metastasis, even across tumor types. Although a regular screening protocol may not be warranted due to the low incidence of CNS metastasis from fallopian tube carcinomas, brain imaging may be a valuable addition to systemic staging for patients with *BRCA1* mutant status, particularly with tumor subtypes (e.g., HER2-enriched or triple negative) that are associated with higher incidences of CNS metastasis (5, 30, 33, 34). The current case is demonstrative of the potential benefit of brain imaging prior to the onset of any gross neurological deficits.

## Data Availability Statement

The original contributions presented in the study are included in the article/**Supplementary Materials**, further inquiries can be directed to the corresponding author.

## Ethics Statement

Ethical review and approval was not required for the study on human participants in accordance with the local legislation and institutional requirements. The patients/participants provided their written informed consent to participate in this study. Written informed consent was obtained from the individual for the publication of any potentially identifiable images or data included in this article.

## Author Contributions

All authors participated in the clinical care of the patient and/or drafting of the manuscript.

## Conflict of Interest

The authors declare that the research was conducted in the absence of any commercial or financial relationships that could be construed as a potential conflict of interest.

## Supplementary Material

Supplementary Table 1 Summary of ongoing interventional clinical trials that are recruiting patients with fallopian tube cancer. The trials were generated in “clinicaltrials.gov” using the search term “Fallopian Tube Cancer” with the status of “Recruiting” and “Enrolling, by invitation,” which yielded a total of 105 studies. From this list, trials were selected if they use novel regimens involving drugs and/or biological agents and did not exclude patients with symptomatic central nervous system metastasis. A brief overview of the objectives, treatment modalities, and recruitment criteria are provided, along with the National Clinical Trial (NCT) number, recruitment size (N), and the current phase of the trial.

Supplementary Table 2 Summary of reviewed cases describing CNS metastasis from a primary fallopian carcinoma. HA, headache; R/L, right/left; IV, intravenous; CNS, central nervous system.

## References

[1] Gavrilovic IT Posner JB. Brain metastases: epidemiology and pathophysiology. J Neurooncol. (2005) 75:5–14. 10.1007/s11060-004-8093-6 16215811

[2] Wethington SL Herzog TJ Seshan VE Seshan VE Bansal N Schiff PB. Improved survival for fallopian tube cancer: a comparison of clinical characteristics and outcome for primary fallopian tube and ovarian cancer. Cancer. (2008) 113:3298–306. 10.1002/cncr.23957 19006196

[3] Eisenhauer EA Therasse P Bogaerts J Sargent D Ford R Dancey J. New response evaluation criteria in solid tumours: revised RECIST guideline (version 1.1). Eur J Cancer. (2009) 45:228–47. 10.1016/j.ejca.2008.10.026 19097774

[4] Liao C-I Chow S Chen L Kapp DS Mann A Chan JK. Trends in the incidence of serous fallopian tube, ovarian, and peritoneal cancer in the US. Gynecol Oncol. (2018) 149:318–23. 10.1016/j.ygyno.2018.01.030 29514737

[5] Cagney DN Martin AM Catalano PJ Redig AJ Lin NU Lee EQ. Incidence and prognosis of patients with brain metastases at diagnosis of systemic malignancy: a population-based study. Neuro Oncol. (2017) 19:1511–21. 10.1093/neuonc/nox077 28444227 PMC5737512

[6] Raghavan D Ahluwalia MS Blanke CD Brown J Kim ES Reaman GH. Textbook of Uncommon Cancer. Oxford: John Wiley & Sons (2017).

[7] Baekelandt M Nesbakken AJ Kristensen GB Tropé CG Abeler VM. Carcinoma of the fallopian tube: clinicopathologic study of 151 patients treated at the Norwegian Radium Hospital. Cancer. (2000) 89:2076–84. 10.1097/00006254-200103000-00015 11066048

[8] Klein M Rosen A Lahousen M Graf A Vavra N Beck A. Lymphogenous metastasis in the primary carcinoma of the fallopian tube. Gynecol Oncol. (1994) 55:336–8. 10.1006/gyno.1994.1302 7835770

[9] Rosen A Klein M Lahousen M Graf AH Rainer A Vavra N. Primary carcinoma of the fallopian tube-a retrospective analysis of 115 patients. Br J Cancer. (1993) 68:605. 10.1038/bjc.1993.394 8353051 PMC1968398

[10] Cormio G Gabriele A Maneo A Bonazzi C Pellegrino A Landoni F. Brain metastases from a primary carcinoma of the fallopian tube. Gynecol Obstet Invest. (1996) 41:286–8. 10.1159/000292286 8793502

[11] Batson OV. The function of the vertebral veins and their role in the spread of metastases. Ann Surg. (1940) 112:138. 10.1097/00000658-194007000-00016 17857618 PMC1387927

[12] Hidaka T Nakamura T Shima T Sumiya S Saito S. Cerebral metastasis from a primary adenocarcinoma of the fallopian tube. Gynecol Oncol. (2004) 95:260–3. 10.1016/j.ygyno.2004.06.036 15385143

[13] Jayashree K Anubuti C Gundappa M. Primary fallopian tube adenocarcinoma with brain and lung metastasis. Indian J Pathol Microbiol. (2009) 52:596. 10.4103/0377-4929.56148 19805996

[14] Merimsky O Inbar M Groswasser-Reider I Neudorfer M Chaitchik S. Sphenoid and cavernous sinuses involvement as first site of metastasis from a fallopian tube carcinoma. Tumori. (1993) 79:444–6. 10.1177/030089169307900615 8171748

[15] Newton HB Stevens C Santi M. Brain metastases from fallopian tube carcinoma responsive to intra-arterial carboplatin and intravenous etoposide: a case report. J Neurooncol. (2001) 55:179–84. 10.1023/A:1013811612546 11859973

[16] Raff JP Anderson P Sands C Makower D. Fallopian tube carcinoma presenting with a brain metastasis. Gynecol Oncol. (2002) 85:372–5. 10.1006/gyno.2002.6595 11972403

[17] Ryuko K Iwanari O Abu-Musa A Fujiwaki R Kitao M. Primary clear cell adenocarcinoma of the fallopian tube with brain metastasis: a case report. Asia Oceania J Obstet Gynaecol. (1994) 20:135–40. 10.1111/j.1447-0756.1994.tb00439.x 8092957

[18] Young JA Kossman CR Green MR. Adenocarcinoma of the fallopian tube: report of a case with an unusual pattern of metastasis and response to combination chemotherapy. Gynecol Oncol. (1984) 17:238–40. 10.1016/0090-8258(84)90082-9 6546733

[19] Jacus MO Daryani VM Harstead KE Patel YT Throm SL Stewart CF. Pharmacokinetic properties of anticancer agents for the treatment of central nervous system tumors: update of the literature. Clin Pharmacokinet. (2016) 55:297–311. 10.1007/s40262-015-0319-6 26293618 PMC4761278

[20] Mahajan A Ahmed S McAleer MF Weinberg JS Li J Brown P. Post-operative stereotactic radiosurgery versus observation for completely resected brain metastases: a single-centre, randomised, controlled, phase 3 trial. Lancet Oncol. (2017) 18:1040–8. 10.1016/S1470-2045(17)30414-X 28687375 PMC5560102

[21] Brown PD Ballman KV Cerhan JH Anderson SK Carrero XW Whitton AC. Postoperative stereotactic radiosurgery compared with whole brain radiotherapy for resected metastatic brain disease (NCCTG N107C/CEC·3): a multicentre, randomised, controlled, phase 3 trial. Lancet Oncol. (2017) 18:1049–60. 10.1016/S1470-2045(17)30441-2 28687377 PMC5568757

[22] US Preventive Services Task Force. Screening for breast cancer: US Preventive Services Task Force recommendation statement. Ann Intern Med. (2009) 151:716. 10.7326/0003-4819-151-10-200911170-00008 19920272

[23] Scheuer L Kauff N Robson M Kelly B Barakat R Satagopan J. Outcome of preventive surgery and screening for breast and ovarian cancer in BRCA mutation carriers. J Clin Oncol. (2002) 20:1260–8. 10.1200/JCO.2002.20.5.1260 11870168

[24] Saslow D Boetes C Burke W Harms S Leach MO Lehman CD. American Cancer Society guidelines for breast screening with MRI as an adjunct to mammography. CA Cancer J Clin. (2007) 57:75–89. 10.3322/canjclin.57.2.75 17392385

[25] Le-Petross HT Whitman GJ Atchley DP Yuan Y Gutierrez-Barrera A Hortobagyi GN. Effectiveness of alternating mammography and magnetic resonance imaging for screening women with deleterious BRCA mutations at high risk of breast cancer. Cancer. (2011) 117:3900–7. 10.1002/cncr.25971 21365619

[26] Thompson D Easton DF. Cancer incidence in BRCA1 mutation carriers. J Natl Cancer Inst. (2002) 94:1358–65. 10.1093/jnci/94.18.1358 12237281

[27] Brose MS Rebbeck TR Calzone KA Stopfer JE Nathanson KL Weber BL. Cancer risk estimates for BRCA1 mutation carriers identified in a risk evaluation program. J Natl Cancer Inst. (2002) 94:1365–72. 10.1093/jnci/94.18.1365 12237282

[28] Berek JS Chalas E Edelson M Moore DH Burke WM Cliby WA. Prophylactic and risk-reducing bilateral salpingo-oophorectomy: recommendations based on risk of ovarian cancer. Obstet Gynecol. (2010) 116:733–43. 10.1097/AOG.0b013e3181ec5fc1 20733460

[29] Ratner E Bala M Louie-Gao M Aydin E Hazard S Brastianos PK. Increased risk of brain metastases in ovarian cancer patients with BRCA mutations. Gynecol Oncol. (2019) 153:568–73. 10.1016/j.ygyno.2019.03.004 30876674

[30] Zavitsanos PJ Wazer DE Hepel JT Wang Y Singh K Leonard KL. BRCA1 mutations associated with increased risk of brain metastases in breast cancer. Am J Clin Oncol. (2018) 41:1252–6. 10.1097/COC.0000000000000466 29782359

[31] Zavitsanos PJ Wazer DE Hepel JT Leonard KL. BRCA1 mutations associated with increased risk of brain metastases in breast cancer: a 2: 1 matched-pair analysis. Int J Radiat Oncol Biol Phys. (2016) 96:S59–60. 10.1016/j.ijrobp.2016.06.153 29782359

[32] Song Y Barry WT Seah DS Tung NM Garber JE Lin NU. Patterns of recurrence and metastasis in BRCA1/BRCA2-associated breast cancers. Cancer. (2020) 126:271–80. 10.1002/cncr.32540 31581314 PMC7003745

[33] Hung MH Liu CY Shiau CY Hsu CY Tsai YF Wang YL. Effect of age and biological subtype on the risk and timing of brain metastasis in breast cancer patients. PLoS ONE. (2014). 10.1371/journal.pone.0089389 24586742 PMC3933537

[34] Cagney DN Martin AM Catalano PJ Brown PD Alexander BM Lin NU. Implications of screening for brain metastases in patients with breast cancer and non-small cell lung cancer. JAMA Oncol. (2018) 4:1001–3. 10.1001/jamaoncol.2018.0813 29799956 PMC6145731

