# Supplementary material for: Case Report: Frontoparietal Metastasis From a Primary Fallopian Tube Carcinoma
Source: Front Surg. 2021 Feb 17;8:594570. doi: 10.3389/fsurg.2021.594570 (PMC7927667; doi:10.3389/fsurg.2021.594570)
Supplement: Supplementary Table 1 — Summary of ongoing interventional clinical trials that are recruiting patients with fallopian tube cancer. The trials were generated in “clinicaltrials.gov” using the search term “Fallopian Tube Cancer” with the status of “Recruiting” and “Enrolling, by invitation,” which yielded a total of 105 studies. From this list, trials were selected if they use novel regimens involving drugs and/or biological agents and did not exclude patients with symptomatic central nervous system metastasis. A brief overview of the objectives, treatment modalities, and recruitment criteria are provided, along with the National Clinical Trial (NCT) number, recruitment size (N), and the current phase of the trial. [file Table_1.DOCX]

**Supplemental Table 1**

| NCT Identifier | Objective | Phase | N | Treatment | Disease criteria |
| --- | --- | --- | --- | --- | --- |
| NCT02948426 | Test the efficacy of IFN stimulated monocytes, with Sylatron and Actimmune in killing tumor cells. | 1 | 40 | Biological: Autologous Monocytes + ACTIMMUNE + SYLATRON | Recurrent or refractory ovarian cancer, fallopian tube cancer or primary peritoneal cancer. |
| NCT02312245 | Determine the response rate of Avatar-directed salvage chemotherapy in patients with platinum-resistant ovarian, primary peritoneal and fallopian tube cancers. | 2 | 240 | Biological: Bevacizumab  Drug: Gemcitabine Hydrochloride  Drug: Paclitaxel  Drug: Pegylated Liposomal Doxorubicin Hydrochloride  Drug: Topotecan Hydrochloride | Platinum-resistant ovarian, primary peritoneal and fallopian tube cancers. |
| NCT03283943 | Test the safety and tolerability of 2 different dose levels of focal sensitizing radiation therapy given with durvalumab. | 1 | 22 | Radiation: Focal radiotherapy  Drug: Durvalumab | Platinum-resistant ovarian, primary peritoneal or fallopian tube epithelial carcinoma. |
| NCT03586661 | Study the best dose and side effects of niraparib and copanlisib in treating patients with recurrent endometrial, ovarian, primary peritoneal, or fallopian tube cancer that has come back. | 1b | 44 | Drug: Copanlisib  Drug: Niraparib | Recurrent high-grade serous or BRCA mutant ovarian cancer or recurrent endometrial, fallopian tube, or primary peritoneal cancer. |
| NCT03585764 | Establish safety and feasibility of intraperitoneally administered lentiviral transduced MOv19-BBz CAR T cells with or without cyclophosphamide + fludarabine as lymphodepleting chemotherapy. | 1 | 18 | Drug: MOv19-BBz CAR T cells  Device: Alpha Folate Receptor expression test | AFR-expressing recurrent high grade serous ovarian, fallopian tube, or primary peritoneal cancer. |
| NCT03213964 | Determine the maximum tolerated dose/maximum feasible dose (MTD/MFD) of a single infusion of FATE-NK100 via intra-peritoneal catheter in women with recurrent ovarian, fallopian tube or primary peritoneal cancer. | 1 | 16 | Biological: FATE-NK100  Drug: Interleukin-2 | Recurrent epithelial ovarian cancer, fallopian tube, or primary peritoneal cancer. |
| NCT03983226 | Evaluate the role of cytoreductive surgery and Niraparib maintenance in platinum-sensitive secondary recurrent ovarian cancer. | 2 | 96 | Procedure: Surgery  Drug: carboplatin/taxane, carboplatin/gemcitabine, cisplatin/gemcitabine, liposome doxorubicin/carboplatin  Drug: Niraparib | Platinum-sensitive, secondary relapsed epithelial ovarian, primary peritoneal, or fallopian tube cancer. |
| NCT03657043 | Study the side effects and efficacy of tisotumab vedotin for platinum-resistant ovarian cancer. | 2 | 182 | Drug: tisotumab vedotin | Platinum-resistant ovarian cancer (PROC) and fallopian tube cancer. |
| NCT03373058 | Test the safety and efficacy of hyperthermic intraperitoneal chemotherapy in the treatment of advanced-stage epithelial ovarian cancer after cytoreductive surgery. | 3 | 310 | Procedure: Hyperthermic Intraperitoneal Chemotherapy  Procedure: cytoreductive surgery  Drug: adjuvant chemotherapy | Primary epithelial ovarian cancer, tubal cancer, and primary peritoneal cancer (Stage III). |
| NCT03056833 | Test whether concurrent ribociclib treatment will enhance the treatment effects of platinum-based chemotherapy for platinum-sensitive recurrent ovarian, fallopian or primary peritoneal cancer. | 1 | 42 | Drug: ribociclib  Drug: Paclitaxel  Drug: Carboplatin | Platinum-sensitive recurrent ovarian, fallopian or primary peritoneal cancer eligible to receive platinum-based doublet chemotherapy. |
| NCT03054909 | Test the efficacy of IL-15Rα-Fc super-agonist complex (ALT-803) given as maintenance therapy after the completion of 1st line IV/IP chemotherapy for the treatment of advanced ovarian, fallopian tube, and primary peritoneal cancer. | 1 | 28 | Biological: ALT-803 Subcutaneous  Biological: ALT-803 Intraperitoneal | Diagnosis of FIGO stage III or grade IV epithelial ovarian, fallopian tube or primary peritoneal carcinoma, has received at least 3 cycles of first line IV/IP cisplatin and paclitaxel chemotherapy and has stable disease or better. |
| NCT02884648 | Learn if Avastin (bevacizumab) can help to control ovarian, fallopian, or primary peritoneal cancer that has been found during second-look surgery. | 2 | 35 | Drug: Bevacizumab | Histologically confirmed Stage III-IV high-grade epithelial non-mucinous ovarian, fallopian tube, or primary peritoneal cancers. |
| NCT03539406 | Study the efficacy of ex vivo-generated allogeneic natural killer (NK) cells with or without preceding non-myeloablative conditioning chemotherapy for recurrent ovarian cancer. | 1 | 12 | Biological: UCB-NK cells  Drug: Chemotherapy | Second recurrence of ovarian, fallopian tube or primary peritoneal cancer. |
| NCT03849469 | Define a maximum tolerated dose and/or recommended dose of XmAb22841 monotherapy and in combination with pembrolizumab in patients with select advanced solid tumors. | 1 | 242 | Biological: XmAb®22841  Biological: Pembrolizumab (Keytruda®) | Histologically or cytologically confirmed advanced or metastatic solid tumors. |
| NCT02433626 | Assess the safety and tolerability of COTI-2 monotherapy or combination therapy in patients with advanced and recurrent malignancies to establish a recommended Phase 2 dose (RP2D) for future studies. | 1 | 51 | Drug: COTI2  Drug: Cisplatin | Cancer that is recurrent, metastatic, or unresectable and for which no effective or curative measures exist. |
| NCT02584478 | Evaluate the safety and efficacy of adding oral AL3818 to standard platinum-based chemotherapy concurrently and continued as a maintenance therapy for up to 12 months. | 1b/2a | 48 | Drug: AL3818  Drug: Carboplatin  Drug: Paclitaxel | Recurrent or metastatic endometrial, ovarian, fallopian, primary peritoneal, or cervical carcinoma. |
| NCT04034927 | Study the efficacy of olaparib with or without tremelimumab in treating patients with recurrent ovarian, fallopian tube, or peritoneal cancer that has come back. | 2 | 170 | Drug: Olaparib  Biological: Tremelimumab | Platinum-sensitive, recurrent high-grade serous or high-grade endometrioid ovarian, primary peritoneal, or fallopian tube cancer. |
| NCT03621982 | Evaluate the safety and efficacy of ADCT-301 in patients with Selected Advanced Solid Tumors. | 1 | 76 | Drug: ADCT-301 | Pathologic diagnosis of solid tumor malignancy that is locally advanced or metastatic at time of screening. |
| NCT03748186 | Study the safety, pharmacokinetics and preliminary efficacy of STRO-002 given intravenously every 3 weeks. | 1 | 160 | Drug: STRO-002 | Advanced epithelial ovarian cancer (including fallopian or primary peritoneal cancer) and endometrial cancer. |
| NCT03587311 | Study the side effects of bevacizumab and anetumab ravtansine or paclitaxel in treating participants with ovarian, fallopian tube, or primary peritoneal cancer that does not respond to treatment. | 2 | 96 | Biological: Anetumab Ravtansine  Biological: Bevacizumab  Drug: Paclitaxel | Histologically or cytologically confirmed high grade serous or high grade endometrioid ovarian, fallopian tube, primary peritoneal cancer. |
| NCT02659384 | Evaluate the efficacy and safety of 5 different treatments involving atezolizumab, bevacizumab and/or acetylsalicylic acid in advanced recurrent platinum-resistant ovarian cancer patients in order to select the optimal treatments for further development in Phase III. | 2 | 160 | Drug: Bevacizumab  Drug: atezolizumab  Drug: acetylsalicylic acid  Drug: placebo | Recurrent, histologically proven, platinum-resistant, epithelial ovarian, fallopian tube and primary peritoneal cancer in advanced or metastatic stage. |
