# Supplementary material for: Case Report: Frontoparietal Metastasis From a Primary Fallopian Tube Carcinoma
Source: Front Surg. 2021 Feb 17;8:594570. doi: 10.3389/fsurg.2021.594570 (PMC7927667; doi:10.3389/fsurg.2021.594570)
Supplement: Supplementary Table 2 — Summary of reviewed cases describing CNS metastasis from a primary fallopian carcinoma. HA, headache; R/L, right/left; IV, intravenous; CNS, central nervous system. [file Table_2.DOCX]

**Supplementary Table 2**

| **Reference** | **Age on CNS diagnosis** | **Time from primary cancer to CNS metastasis** | **Presenting symptoms** | **Metastatic lesion** | **Treatment for metastatic tumor** |
| --- | --- | --- | --- | --- | --- |
| Cormio et al., 1996 ^10^ | 65 | 3 years | HA, dizziness | R cerebellum | Whole-brain radiotherapy, supportive therapy |
|  | 52 | 3 years | HA, stumbling, R hemiparesis | Multiple lesions in L hemisphere | Steroids, supportive therapy |
|  | 61 | 4 years | HA, R hemiparesis | Multiple lesions in L hemisphere | Whole-brain radiotherapy |
| Young et al., 1984 ^17^ | 53 | 3 years | Numbness/tingling of L face, episode of dysphasia | Not reported* | Whole-brain radiotherapy |
| Merimsky et al., 1993 ^13^ | 77 | 3 months | Left facial numbness, L palpebral ptosis, diplopia, retro-ocular pain | L sphenoid sinus, L cavernous sinus | Partial resection |
| Ryuko et al., 1994 ^16^ | 61 | 52 months | Vertigo,  headache, vomiting, gait disturbances | Cerebellum | Whole-brain radiotherapy and steroids |
| Raff et al., 2002 ^15^ | 62 | Unknown | L hemiparesis | R parietal lobe | Resection, whole-brain radiotherapy |
| Hidaka et al., 1998 ^11^ | 61 | 2 years | severe HA, dizziness, nausea, and vomiting. L homonymous hemianopsia and hemiplegia | R occipital lobe | Resection, five cycles of platinum-based combination chemotherapy |
| Newton et al., 2001 ^14^ | 48 | 1 year | HA, visual difficulties, persistent nausea and emesis, gait imbalance | Brainstem, R temporal lobe | Intra-arterial carboplatin and IV etoposide |
| Jayashree et al., 2009 ^12^ | 58 | Unknown | HA, disarticulation and dysphagia | Multifocal, R-sided skull-base | Not reported |
